# Risk of re-identification of epigenetic methylation data: a more nuanced response is needed

**DOI:** 10.1186/s13148-015-0079-z

**Published:** 2015-04-18

**Authors:** Yann Joly, Stephanie OM Dyke, Warren A Cheung, Mark A Rothstein, Tomi Pastinen

**Affiliations:** McGill Epigenome Mapping Centre, Centre of Genomics and Policy, Faculty of Medicine, McGill University, 740 Dr. Penfield Avenue, Montreal, Quebec H3A 0G1 Canada; McGill Epigenome Mapping Centre, Faculty of Medicine, McGill University and Genome Quebec Innovation Centre, 740 Dr. Penfield Avenue, Montreal, Quebec H3A 0G1 Canada; Institute for Bioethics, Health Policy and Law, University of Louisville School of Medicine, 501 East Broadway, Suite #310, Louisville, KY 40292 USA

**Keywords:** Privacy, Data sharing, Epigenome, Policy

## Abstract

In this letter to the editor, we respond to the recent publication by Philibert *et al. Methylation array data can simultaneously identify individuals and convey protected health information: an unrecognized ethical concern* (Clinical Epigenetics 2014, 6:28). Further discussion of the issues raised by the risk of re-identification of epigenetic methylation data is needed, and a more nuanced approach should be taken with respect to its implications for data sharing policy than the one provided.

We welcome the recent publication on the risk of re-identification of methylation array data by Philibert *et al*. [1], as it raises important questions concerning access to epigenetic data which we are carefully considering as members of the International Human Epigenome Consortium (IHEC). While sensitive to the importance of protecting research participants’ identifiable health data, we believe that ultimately the adequate level of protection should be determined by taking into account the scientific, social, and policy context and following a thorough risk-benefit analysis of the research being undertaken. In light of this, we would like to express certain reservations regarding the analysis and findings presented in this paper.

We have some reservations regarding the authors’ statement that ‘there was an erroneous expectation that anonymized genome-wide genetic data contained within repositories could not be linked to identifiable individuals’. This assumption, according to the authors, led to the unsound belief that information contained within publicly available data cannot be used to both infer disease status and uniquely identify individuals. While we agree that the limits of data anonymization are now better understood following numerous research papers on this topic, it should be understood that most of this research refers to hypothetical scenarios leading to assessments of low risk of re-identification [2,3]. Moreover, as the authors recognize: ‘with the exception of isolated instances, protected information regarding disease status has not been compromised’. For these reasons, apart from one instance following a publication by Homer *et al*. [4], the scientific and policy community generally has not chosen to increase the level of protection of genome-wide genetic data by restricting its access through controlled access administrative processes [5].

Furthermore, Philibert *et al*. limit their study of genotype to a single cell type (from peripheral blood). Several studies have also already identified genetic polymorphisms directly affecting methylation data [6,7]. Moreover, the form of re-identification discussed by Philibert *et al*. would require the individual’s genetic data, in which case the information at risk is not the genetic data but any associated patient or participant information. It is nevertheless important to consider what additional health information could be revealed by epigenetic information. The ‘imputation of phenotypic data’ from methylomes is valid (replicated) for smoking and blood methylomes, but for any other trait, we do not currently have unequivocal evidence of health or exposure data being easily read from these data. Results shown by Figure one in Philibert *et al*. show far from perfect prediction, even given homogeneous sampling to call differences between smokers and nonsmokers in a controlled experiment. The result is not unexpected since recent studies [8] have shown a complex relationship with blood methylation and smoking, where the intensity of exposure is poorly correlated with most changes and some sites revert to ‘normal’ methylation following cessation, whereas others persist. Similarly, for gene expression datasets, and using pre-existing genetic and expression data from matching tissue and processing techniques, individual genetic variation can be predicted from its impact on gene expression [9]. Furthermore, blood transcriptomes can reflect smoking just as methylomes do [8]. Consequently, many risks reported by the authors would be comparable to those for gene expression arrays. In the case of both methylation and gene expression data, the risk of re-identification relies on access to the DNA of the research subject and with such biospecimens many similarly ‘imputable’ phenotypes become accessible. We note that microarray-based techniques are being replaced by next-generation sequencing methods in large-scale epigenome mapping efforts such as IHEC, and will likely penetrate to cohort studies, given the approximately 50-fold higher information content of next-generation sequencing data as compared to Illumina 450K methylation variants with ‘high accuracy phenotypic imputation’ that may emerge, and obviously, this data provides unique challenges regarding genotypic data privacy.

Our major point of contention with Philibert *et al*. concerns their conclusion that a preferred response to their findings would be that ‘access to genome methylation data be restricted to institutionally approved investigators who accede to data agreements prohibiting re-identification’. Although IHEC is deeply committed to the safeguarding of sensitive health information, we believe this proposal is premature and an overreaction for the following reasons: 1. as noted, the data analysis is based on technology that is being superseded by next-generation sequencing; 2. the risk of re-identification is remote; 3. the health information currently at risk consists of tobacco and alcohol usage - this information is widely available in medical records and nonmedical sources; 4. going forward, more comprehensive measures need to be developed that consider prospective informed consent for this type of research; and 5. the proposal to limit access could add to the burden of institutional review boards and unnecessarily impede research.

Ideally, re-identification research should consider not only the technical potential to achieve re-identification but also the full spectrum of administrative, legal (which in the U.S. is not limited to HIPAA), and information technology measures available to reduce the existing risk. Once this is done, a careful risk-benefit analysis will need to be undertaken. In this analysis, the benefit of broad data sharing to medical research, and sometimes directly to participants (for example, through return of clinically significant results), should not be underestimated [10]. While several aspects of the consent process eventually need to be revisited to be adapted to reflect current technological and scientific practices, shifting the discussion surrounding informed consent from unrealistic promises of confidentiality protection towards a greater focus on transparency and clarity regarding the risk actually incurred by participants in OMICS data sharing projects would be a useful response for all involved. To better communicate this information to participants, it could be worth contrasting the risk incurred by participants accepting the open release of their genetic expression data to that incurred in everyday life by a regular Internet user.

## Abbreviations

HIPAA: Health Insurance Portability and Accountability Act
IHEC: International Human Epigenome Consortium
